# Risk Stratification of COVID-19 Patients Using Ambulatory Oxygen Saturation in the Emergency Department

**DOI:** 10.5811/westjem.2020.8.48701

**Published:** 2020-09-24

**Authors:** Arvin R. Akhavan, Joseph P. Habboushe, Rajneesh Gulati, Oluchi Iheagwara, Joanna Watterson, Shawn Thomas, Jordan L. Swartz, Christian A. Koziatek, David C. Lee

**Affiliations:** * New York University Grossman School of Medicine, Ronald O. Perelman Department of Emergency Medicine, New York, New York; † New York University Grossman School of Medicine, Department of Population Health, New York, New York

## Abstract

**Introduction:**

It is difficult to determine illness severity for coronavirus disease 2019 (COVID-19) patients, especially among stable-appearing emergency department (ED) patients. We evaluated patient outcomes among ED patients with a documented ambulatory oxygen saturation measurement.

**Methods:**

This was a retrospective chart review of ED patients seen at New York University Langone Health during the peak of the COVID-19 pandemic in New York City. We identified ED patients who had a documented ambulatory oxygen saturation. We studied the outcomes of high oxygen requirement (defined as >4 liters per minute) and mechanical ventilation among admitted patients and bounceback admissions among discharged patients. We also performed logistic regression and compared the performance of different ambulatory oxygen saturation cutoffs in predicting these outcomes.

**Results:**

Between March 15–April 14, 2020, 6194 patients presented with fever, cough, or shortness of breath at our EDs. Of these patients, 648 (11%) had a documented ambulatory oxygen saturation, of which 165 (24%) were admitted. Notably, admitted and discharged patients had similar initial vital signs. However, the average ambulatory oxygen saturation among admitted patients was significantly lower at 89% compared to 96% among discharged patients (p<0.01). Among admitted patients with an ambulatory oxygen saturation, 30% had high oxygen requirements and 8% required mechanical ventilation. These rates were predicted by low ambulatory oxygen saturation (p<0.01). Among discharged patients, 50 (10%) had a subsequent ED visit resulting in admission. Although bounceback admissions were predicted by ambulatory oxygen saturation at the first ED visit (p<0.01), our analysis of cutoffs suggested that this association may not be clinically useful.

**Conclusion:**

Measuring ambulatory oxygen saturation can help ED clinicians identify patients who may require high levels of oxygen or mechanical ventilation during admission. However, it is less useful for identifying which patients may deteriorate clinically in the days after ED discharge and require subsequent hospitalization.

## INTRODUCTION

One of the most difficult challenges in the management of coronavirus 2019 (COVID-19) patients is identifying those with significant respiratory compromise.^1^,^2^ Some patients without any visible respiratory distress can have severe hypoxemia, and there is substantial variability in the severity of illness among COVID-19 patients.^3^–^5^ Therefore, there is a significant amount of uncertainty surrounding the care of these patients, particularly with regard to disposition from the emergency department (ED).^6^ These decisions are made more complicated by the life-threatening nature of this illness and the massive burden that COVID-19 has placed on an already strained healthcare system.^7^–^9^

As the pandemic has evolved, several studies have identified patient characteristics and clinical markers that are correlated with poor outcomes among COVID-19 patients.^10^–^12^ Many of these studies use an outcome of intubation or death to risk stratify patients. However, there are COVID-19 patients who will develop high oxygen requirements and may require admission to avoid these endpoints.^13^–^16^ The criteria used to determine which ED patients should be admitted may not be the same as those factors that predict intubation or death. Furthermore, in the face of overwhelming patient volumes, many ED clinicians may find that they lack the capacity to perform comprehensive laboratory or radiologic testing on all patients presenting with COVID-19 symptoms.^17^,^18^

During the surge of ED patients in New York City, ED clinicians (physicians, residents, and physician assistants) at our institution developed the practice of performing ambulatory oxygen saturation measurements to aid the disposition of stable-appearing COVID-19 patients. Previously, oxygen desaturation while walking has been shown to be associated with poor outcomes in diseases such as pulmonary fibrosis and radiation pneumonitis.^19^–^22^ The goal of this study was to provide data on our early experience using ambulatory oxygen saturation to determine whether this relatively quick assessment can help guide the disposition of ED patients with COVID-19.

## METHODS

### Study Design, Setting, and Population

We performed a retrospective chart review of ED patients seen at New York University (NYU) Langone Health at our four EDs, located in Manhattan, Brooklyn, and Long Island. We studied ED visits during the month (specific dates below) that corresponded to the peak of the COVID-19 pandemic in New York State. Charts were reviewed to identify ED patients who had a documented oxygen saturation while ambulating. We then analyzed the association between recorded ambulatory oxygen saturation and patient outcomes among patients admitted and discharged from the ED.

**Population Health Research Capsule**

What do we already know about this issue?

*The COVID-19 pandemic is rapidly evolving, and little is known about the ability to risk stratify patients based on ambulatory oxygen saturation*.

What was the research question?

*Can ambulatory oxygen (O_2_ sat) saturation help guide disposition of emergency department (ED) patients with COVID-19?*

What was the major finding of the study?

*Ambulatory O_2_ sat cannot rule out ED bounceback to admission, but does predict inpatient respiratory needs*.

How does this improve population health?

*At the pandemic’s height, EDs lacked evidence-based ways to quickly risk stratify respiratory patients. This study provides early data for one approach*.

### Data Sources

We queried the health network’s electronic health record (EHR) (Epic Systems, Verona, WI) via Oracle SQL Developer (Oracle Corporation, Redwood Shores, CA) in our Epic Systems Clarity database. We exported initial ED clinician notes along with demographic variables (ie, age and gender) and clinical variables (ie, body mass index [BMI], medical comorbidities, and initial ED vital signs) for all ED patients presenting with COVID-19 symptoms from March 15, 2020–April 14, 2020. In addition, we abstracted additional clinical outcomes (.e, supplemental oxygen flow rates and devices and bounceback admissions to our facilities) for confirmed COVID-19 positive patients admitted as inpatients to the hospital from the ED. We performed data abstraction on April 29, 2020, to ensure that at least two weeks of outcome data were available for each patient.

### Ambulatory Oxygen Saturation

When the initial ED clinician note for a patient contained the key words walk/walked/walking or “ambul” to capture ambulatory/ambulation/ambulated/ambulating, we reviewed the chart to determine whether a numeric ambulatory oxygen saturation had been documented in any of the ED notes. When a range of values was charted, we used the lowest number. In several cases, ED clinicians noted that the patient’s oxygen saturation while walking was greater than some number (eg, “>93%”). When we asked our ED clinicians, the consensus was that this should be interpreted to mean equal to or greater than that number as it is difficult to type a greater than or equal to sign in the EHR. In a minority of cases, ED clinicians wrote partially numeric values (eg, “high 80s” or “mid 90s”). These values were reinterpreted as follows: high 90s (two instances of this phrase assigned 98%); low-mid 90s (one assigned 93%); low 90s (two assigned 92%,); high 80s (five assigned 88%); mid-high 80s (one assigned 87%); mid 80s (three assigned 85%); low 80s (four assigned 82%).

### Primary Outcomes

For admitted ED patients, our clinical outcome was a high oxygen requirement, defined as an oxygen flow rate above four liters per minute (L/min) at any point during hospitalization, which included the need for mechanical ventilation. We used this value as a cutoff given that most patients on home oxygen are generally not at rates higher than four L/min. For discharged ED patients, our clinical outcome was bounceback admission, defined as a subsequent ED visit within 10 days of the initial ED visit that resulted in an inpatient hospitalization. Notably, we were not able to track whether a patient had a bounceback admission at other area hospitals.

### Statistical Analysis

We initially described our retrospective cohort of patients who had a documented ambulatory oxygen saturation based on demographic variables, BMI, medical comorbidities, initial ED vital signs, and documented ambulatory oxygen saturation. We analyzed categorical variables by chi-square tests, and continuous variables by t-tests and rank-sum tests as appropriate. A p-value of 0.05 was used to identify statistically significant differences in the characteristics of ED patients with a documented ambulatory oxygen saturation who were admitted vs discharged.

We then analyzed the association between the documented ambulatory oxygen saturation and our clinical outcomes using logistic regression. Since there were two main analyses in this study (among admitted patients and separately among discharged patients), we used a Bonferroni correction and an adjusted p-value of 0.025 to test for a significant association between ambulatory oxygen saturation and our clinical outcomes. Finally, we also analyzed the performance of ambulatory oxygen saturation in terms of sensitivity, specificity, negative predictive value (NPV), and positive predictive value (PPV) at different ambulatory oxygen saturation cutoffs. Statistical analyses were performed in Stata 16.1 (StataCorp, College Station, TX). This study was approved by the institutional review board at NYU Grossman School of Medicine.

## RESULTS

### Study Population

Of the 17,123 ED patients seen at our four EDs in Manhattan, Brooklyn, and Long Island between March 15–April 14, 2020, 6194 (36%) had a chief complaint of either fever, cough, or shortness of breath. Of the patients presenting with these symptoms, 1071 (17%) had the key words: walk, walked, walking, ambulatory, ambulation, ambulated, or ambulating. When we reviewed these charts with the key words present, 684 (64%) had a documented number for an ambulatory oxygen saturation and 165 (24%) of these patients were admitted.

**Figure 1:**
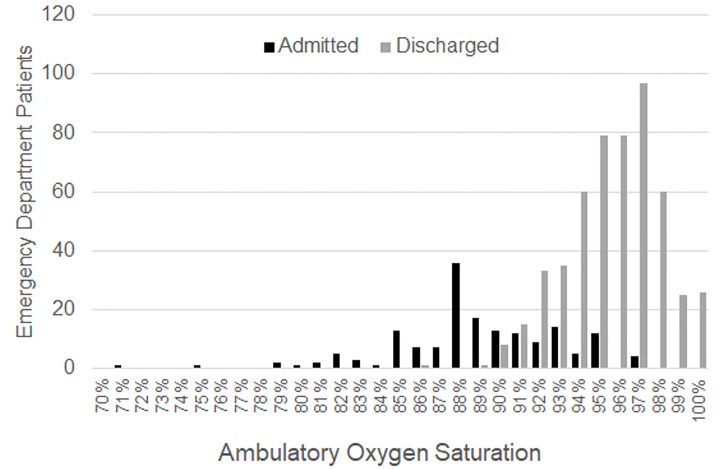
Distribution of documented ambulatory oxygen saturation among admitted and discharged ED Patients

Comparing admitted and discharged ED patients with a documented ambulatory saturation, admitted patients were approximately 10 years older than discharged patients and more frequently had a history of hypertension, hyperlipidemia, diabetes, cirrhosis, or immunosuppression (Table 1). As for initial triage vital signs, there was a statistically significant difference between the initial temperature, diastolic blood pressure, and triage oxygen saturation between admitted and discharged ED patients. In general, these differences in triage vital signs were not necessarily clinically significant. Although the ranges of their initial triage oxygen saturation values were the same, the average and median ambulatory oxygen saturation of discharged ED patients was 96% (range of 86–100%) compared to 89% (range of 71–95%) among admitted ED patients (Figure 1).

### Clinical Outcomes

**Figure 2:**
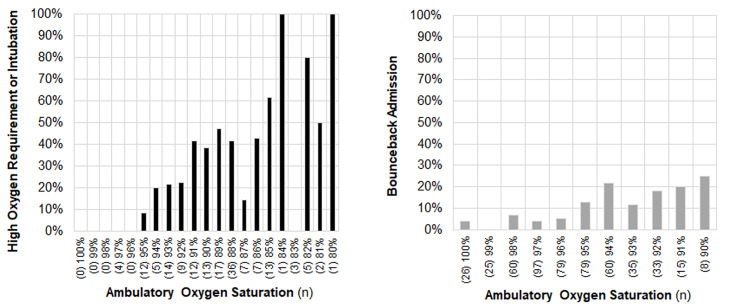
Proportion of emergency department (ED) admissions with high oxygen requirements or intubation and proportion of ED discharges with bounceback admission. Note: Number of patients at each ambulatory oxygen saturation value noted in parentheses.

Of the 165 admitted ED patients with a documented ambulatory oxygen saturation, 103 (62%) did not require more than four L/min of oxygen during their hospitalization, 49 (30%) required more than four L/min of oxygen, and 13 (8%) required mechanical ventilation. Of the 519 discharged ED patients with a documented ambulatory oxygen saturation, 50 (10%) had a subsequent ED visit at our health system that resulted in an inpatient hospitalization, which is higher than our typical bounceback rate or overall bounceback rate during this time period. Of these bounceback admissions, 24 (48%) had a low oxygen requirement, 19 (38%) had a high oxygen requirement, and 7 (14%) required mechanical ventilation. We also stratified these outcomes by different ambulatory oxygen saturation levels in Figure 2 and Table 2.

### Prediction Based on Ambulatory Oxygen Saturation

In our univariable logistic regression analyses, a higher ambulatory oxygen saturation among admitted ED patients was associated with lower odds of high oxygen requirement or mechanical ventilation (p<0.01). Similarly, a higher ambulatory oxygen saturation among discharged ED patients was associated with a lower odds of bounceback admission (p<0.01).

**Figure 3:**
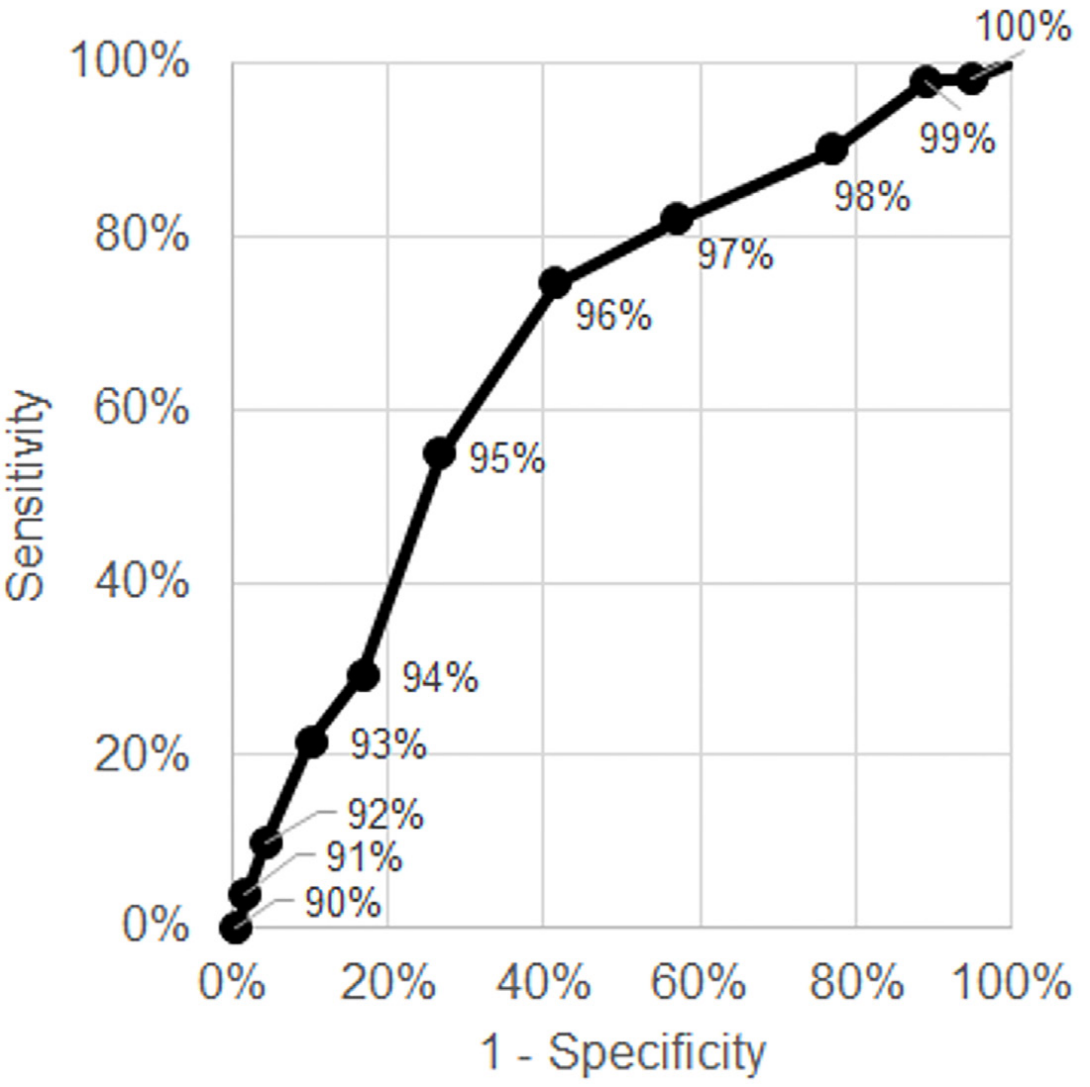
Receiver operating characteristics curve for bounceback admission among discharged emergency department patients.

**Figure 4:**
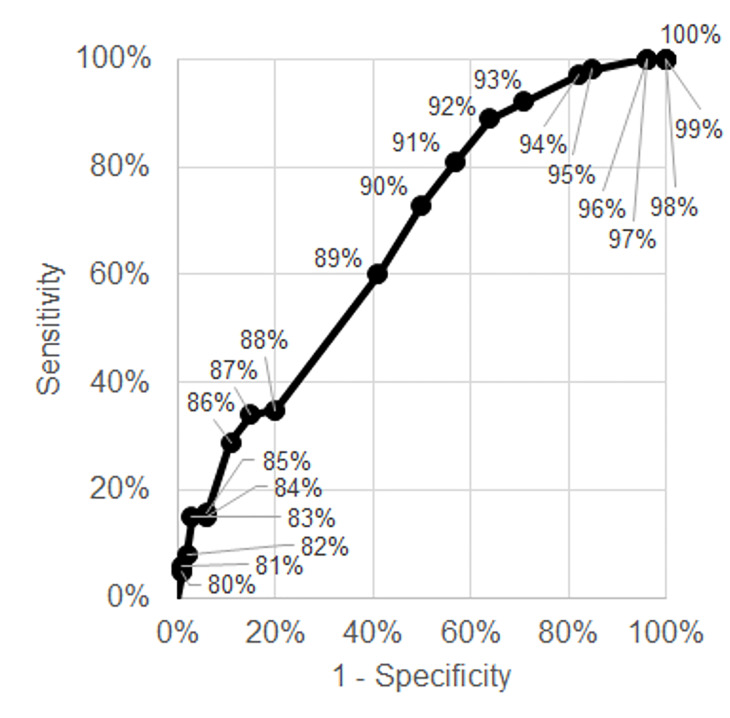
Receiver operating characteristics curve for high oxygen requirement or intubation among admitted emergency department patients.

We also provide a range of performance characteristics (ie, sensitivity, specificity, NPV, and PPV) for different cutoffs for ambulatory oxygen saturation for these outcomes in Table 3, along with receiver operating characteristic curves in Figures 3 and 4. For example, an ambulatory oxygen saturation of 92% or less among admitted ED patients had a 92% sensitivity, 29% specificity, 86% NPV, and 44% PPV for requiring a high level of supplemental oxygen or mechanical ventilation. For discharged patients, even those with high oxygen saturations (up to 98%) on ambulation had a chance of representing with subsequent admission.

## DISCUSSION

Our goal in this study was to evaluate whether the measurement of ambulatory oxygen saturation could help predict outcomes among admitted and discharged ED patients. It should be noted that our study population included only ED patients who were able to tolerate ambulation and therefore likely excludes patients who were critically ill or had a high oxygen requirement at baseline. This study population is critically important to examine since it represents a population of relatively stable-appearing ED patients. Because of the clinical characteristics of COVID-19, it can be difficult to differentiate patients with respiratory compromise given that some patients do not present with increased work of breathing and may appear clinically well.^1^,^2^ In fact, in our study population, the resting vital signs of admitted and discharged ED patients were relatively similar. Ambulatory oxygen saturation values differed between these two groups significantly, which is expected, given that our ED clinicians were making admission decisions based on these values.

In this study, we found that a lower ambulatory oxygen saturation was strongly associated with a requirement of high oxygen supplementation or mechanical ventilation among admitted ED patients. In our study population, no patient with an ambulatory oxygen saturation of 96% or higher required high oxygen supplementation, and no patient 95% or higher required mechanical ventilation during their hospitalization, although it should be noted that our sample of such patients was not large. The proportion of patients who eventually required these treatments appears to increase consistently below these values, especially around 92% and below, which would be consistent with the transition to the steeper portion of the oxyhemoglobin dissociation curve.

Guidelines from the World Health Organization at the time of this publication recommend hospitalization for suspected COVID-19 patients with an oxygen saturation less than or equal to 93%.^23^ This standard applied to only 41% of the patients actually admitted in our study population. This criterion would have had a 55% sensitivity, 68% specificity, 71% NPV, and 51% PPV for high oxygen requirement or mechanical ventilation. In comparison, using only an ambulatory oxygen saturation cutoff of less than or equal to 93%, approximately 87% of the admitted patients would have met this ambulatory oxygen saturation criterion, which would have had a 97% sensitivity, 18% specificity, 90% NPV, and 42% PPV for high oxygen requirement or mechanical ventilation. While there were other factors that determined whether patients in our study population were admitted, it appears that ambulatory oxygen saturation can help identify additional COVID-19 patients who may have poor outcomes and warrant inpatient hospitalization.

Of discharged ED patients with a documented ambulatory oxygen saturation, 9.6% returned to one of our institutions for a subsequent ED visit resulting in hospital admission. Of these patients with a bounceback admission, over 50% required a high level of oxygen or mechanical ventilation. This bounceback admission rate of 9.6% in our study population compares to an overall rate of approximately 1.5% at our institution, which suggests that our study population of patients with a documented ambulatory oxygen saturation was generally a higher risk group even though they did not present critically ill or with an obvious oxygen requirement. It is possible that ED clinicians were more likely to perform an ambulatory oxygen saturation if they thought that the patient was more concerning and wanted additional data to make a disposition decision. Furthermore, we should note that these bounceback admissions were only tracked at our institution and likely underestimate the true bounceback rate, given that patients might have been subsequently admitted to other hospitals.

In this study, we did find that a lower ambulatory oxygen saturation was associated with a higher likelihood of bounceback admission. However, our analysis of the performance of different cutoffs suggests that the ambulatory oxygen saturation would probably not be clinically useful in predicting the future clinical trajectory of patients (eg, only 28% sensitivity and 15% PPV for bounceback admission at an ambulatory oxygen saturation of 93% or less during the first ED visit). In addition, there were discharged ED patients who required high levels of oxygen or mechanical ventilation on a subsequent inpatient hospitalization at a variety of ambulatory oxygen saturation levels at the first ED visit. These findings are likely indicative of the high variability in clinical outcomes among COVID-19 patients and that a single one-time measurement of ambulatory oxygen saturation in isolation will not be able to predict whether a patient will develop worsening respiratory compromise in the days after discharge from the ED. We believe this is an extremely important point for emergency clinicians, given that spikes in respiratory volume during potential future waves of COVID-19 may necessitate simple and quick risk stratification strategies. Ambulatory oxygen saturation, in isolation, does not definitively predict future respiratory compromise given the unpredictable disease course among COVID-19 patients.

We also performed a post-hoc case review of ED patients in our study who had a bounceback admission that resulted in the need for mechanical ventilation. In this analysis, although some patients had a normal ambulatory oxygen saturation, a few of these patients developed some level of tachycardia or tachypnea during ambulation despite maintaining a normal oxygen saturation. In our clinical experience, many of our ED clinicians used these other cues during the measurement of ambulatory oxygen saturation to inform their clinical decision-making. For instance, some patients were admitted if they developed severe tachycardia, exertional lightheadedness, or were otherwise unable to tolerate ambulation during these tests. However, we do not have any data on how well these other factors predict poor outcomes. The reliance on any single number is likely suboptimal compared to its inclusion with a physician’s clinical gestalt and other objective findings.

Measurement of ambulatory oxygen saturation has been used in the evaluation of patients in other disease states, including pulmonary fibrosis and radiation pneumonitis.^19^,^20^ There is some suggestion in the literature that exertional hypoxemia is more commonly a feature of restrictive, rather than obstructive, pulmonary pathology.^24^–^26^ Therefore, the disposition decision for COVID-19 patients with chronic obstructive pulmonary disease (COPD) may require a different set of factors or measures. While the pathophysiology of COVID-19 is still unclear, our study demonstrates that ambulatory oxygen saturation may have some prognostic value among COVID-19 patients.^15^ Some methodological data regarding risk stratification for COVID-19 patients is emerging, but much of it requires additional studies, such as laboratory bloodwork.^27^–^29^ At the height of the pandemic wave in our institution, it would have been nearly impossible to perform this type of risk stratification given the high volume of COVID-19 patients presenting to the ED.

While we provide evidence for the use of ambulatory oxygen saturation among ED patients, we acknowledge that the threshold for admission might depend on a number of factors and may change in different phases of the pandemic depending on the balance between ED patient arrivals and inpatient hospital capacity. Furthermore, among patients who are already hospitalized, the use of ambulatory oxygen saturation to determine when to discharge inpatients may differ from our results given that most of these hospitalized patients have already been through a period of observation in which the patients may have already clinically deteriorated or demonstrated the clinical stability and improvement for a safe inpatient discharge.

Although it might be tempting to apply broad recommendations regarding disposition decisions based on our data, it is important to note that this was a retrospective study, and the characteristics of our hospital system in terms of capacity and patient population may be different from other hospital settings. Hospital guidelines and policies need to consider multiple factors, especially whether there is an ability to send discharged ED patients home with supplemental oxygen and home monitoring or be sent to a lower acuity environment for further observation. Acceptable rates of bounceback admissions and escalation of care are undoubtedly dependent on many factors, particularly in the midst of a pandemic. Therefore, it is probable that some flexibility in the deployment of guidelines on ambulatory oxygen saturation prior to ED disposition would be important as well.

Further research is needed to identify COVID-19 patients who are likely to have poor outcomes with a focus on ED patient populations who appear clinically stable given the difficulty in identifying COVID-19 patients with respiratory compromise. Several research initiatives are trying to develop clinical risk stratification tools, but few focus on the ED and its patient population, even though the ED has been the central point of critical disposition decisions. Abnormal vital signs, patient risk factors, laboratory findings, imaging, and clinical gestalt together inform clinical decision-making. Our study suggests that measuring an ambulatory oxygen saturation can be another tool to support ED clinicians who may face limited data on which to make clinical decisions during this pandemic, but it will not be able to predict all potential decompensations.

## LIMITATIONS

Our study was a retrospective review of patients at a single, large, academic health system during the height of the COVID-19 pandemic. During this period, patients may have been treated in triaged in non-conventional ways. Although our four EDs and three hospitals have different patient populations, our study findings may not be generalizable to ED patient populations at other institutions or areas of the country. Furthermore, there were no standardized protocols in place at our institution for how to use the ambulatory oxygen saturation. Some clinicians may have ambulated their patients for a longer distance or time period and used a different cutoff for disposition decisions, which is reflected in the variation in our study population. In addition, ambulatory oxygen saturation was likely used to risk stratify those who were more ill than the typical well-appearing respiratory patients, which may have introduced a component of selection bias in our cohort of admitted vs discharged patients. The timing of ambulatory oxygen saturation measurement may have been different. Some patients may have been at earlier or later stages of disease, and this may add some uncertainty to the study findings.

Given that our study was retrospective, the use of ambulatory oxygen saturation needs prospective validation. However, this study provides data in a practice environment where front-line healthcare clinicians must make clinical decisions with a paucity of data to support them. Additionally, during this period of peak COVID-19 volume in New York City, hospitals did not have testing capacity to confirm COVID-19 disease in all patients. This allows for the possibility that our outpatient sample may have included other disease processes, such as bacterial pneumonia.

In addition, we do not have data for patients who were subsequently admitted to other hospitals outside our institution; therefore, the rate of bounceback admissions was very likely underestimated. Whereas ambulatory oxygen saturation may identify additional patients who need to be admitted to the hospital, its use alone will definitely not identify all COVID-19 patients who will require a future admission. Statistically, there may have been a non-linear relationship between ambulatory oxygen saturation and our primary outcome, especially given the shape of the oxyhemoglobin dissociation curve. Finally, our retrospective electronic chart abstraction was limited by our search parameters, so charts that included ambulatory oxygen saturation with other unique abbreviations, or an ambulatory saturation documented by other ED staff, may have been missed.

## CONCLUSION

Measuring ambulatory oxygen saturation can help ED clinicians identify patients who may require high levels of oxygen or mechanical ventilation during admission. However, it less useful for identifying which patients may deteriorate clinically in the days after ED discharge and require subsequent hospitalization.

## Figures and Tables

**Table 1:** Characteristics of admitted and discharged ED patients with a documented ambulatory oxygen saturation.

Patient characteristics	Admitted N (%) or Mean (SD)	Discharged N (%) or Mean (SD)	Significance P-value
Total patients	165	519	
Age			
Mean	56	47	< 0.01
Median	58	47	< 0.01
Interquartile range	47 to 66	37 to 57	
18 to 29	5 (3%)	53 (10%)	
30 to 39	17 (10%)	119 (23%)	
40 to 49	30 (18%)	108 (21%)	
50 to 59	36 (22%)	137 (26%)	
60 to 69	51 (31%)	72 (14%)	
70 to 79	23 (14%)	26 (5%)	
80 and up	3 (2%)	4 (1%)	
Sex			
Male	94 (57%)	270 (52%)	0.27
Female	51 (43%)	249 (48%)	
Body-Mass-Index*			
20 to 25	22 (16%)		
25 to 30	53 (39%)		
30 to 35	31 (23%)		
40 to 45	18 (13%)		
45 and Up	4 (9%)		
Comorbidities			
Hypertension	73 (44%)	97 (19%)	< 0.01
Hyperlipidemia	43 (26%)	88 (17%)	0.01
Diabetes	34 (20%)	57 (11%)	< 0.01
Coronary artery disease	7 (4%)	20 (4%)	0.82
Congestive heart failure	2 (1%)	5 (1%)	0.78
Asthma	20 (12%)	42 (8%)	0.12
COPD	1 (1%)	4 (1%)	0.83
Cancer	9 (5%)	28 (5%)	0.98
Cirrhosis	2 (1%)	0 (0%)	0.01
Chronic kidney disease	6 (4%)	13 (3%)	0.44
End-stage renal disease	2 (1%)	2 (0%)	0.23
Immunosuppression	10 (6%)	6 (1%)	< 0.01
Triage vital signs			
Temperature	99.8 (1.5)	99.5 (1.3)	< 0.01
Heart rate	97 (17)	97 (16)	0.58
Systolic blood pressure	132 (18)	132 (17)	0.74
Diastolic blood pressure	77 (11)	81 (11)	< 0.01
Respirations	20 (3)	20 (3)	0.02
Triage oxygen saturation			
Average	95 (2)	97 (2)	< 0.01
Median	95	97	< 0.01
Range	90 to 100	90 to 100	
Ambulatory oxygen saturation			
Average	89 (4)	96 (2)	< 0.01
Median	89	96	< 0.01
Range	71 to 97	86 to 100	< 0.01

*COPD*, Chronic obstructive pulmonary disease.

* Only 24 of the 519 discharged patients had a height and weight measurement to calculate a body-mass-index, therefore these values are not reported.

**Table 2:** Patient outcomes stratified by ambulatory oxygen saturation among admitted and discharged ED patients.

Patient outcome	Admitted on first ED Visit (n = 165)	Bounceback admission (n = 50)
Among all patients		
Low oxygen requirement	103 (62%)	24 (48%)
High oxygen requirement	49 (30%)	19 (38%)
Mechanical ventilation	13 (8%)	7 (14%)
Ambulatory oxygen saturation 98% to 100%		
Low oxygen requirement	0 (0%)	2 (40%)
High oxygen requirement	0 (0%)	2 (40%)
Mechanical ventilation	0 (0%)	1 (20%)
Ambulatory oxygen saturation 95% to 97%		
Low oxygen requirement	15 (94%)	9 (50%)
High oxygen requirement	1 (6%)	6 (33%)
Mechanical ventilation	0 (0%)	3 (17%)
Ambulatory oxygen saturation 93% to 94%		
Low oxygen requirement	15 (79%)	9 (53%)
High oxygen requirement	3 (16%)	7 (41%)
Mechanical ventilation	1 (5%)	1 (6%)
Ambulatory oxygen saturation 90% to 92%		
Low oxygen requirement	22 (65%)	4 (40%)
High oxygen requirement	10 (29%)	4 (40%)
Mechanical ventilation	2 (6%)	2 (20%)
Ambulatory oxygen saturation 89% and below		
Low oxygen requirement	51 (53%)	0 (0%)
High oxygen requirement	35 (37%)	0 (0%)
Mechanical ventilation	10 (10%)	0 (0%)

*ED*, emergency department.

**Table 3:** Performance characteristics of a range of ambulatory oxygen saturation cutoffs among admitted and discharged ED patients.

Ambulatory oxygen saturation	Sensitivity	Specificity	Negative predictive value	Positive predictive value
High oxygen requirement or intubation among admitted ED patients				
95% or less	100%	4%	100%	39%
94% or less	98%	15%	94%	41%
93% or less	97%	18%	90%	42%
92% or less	92%	29%	86%	44%
91% or less	89%	36%	84%	45%
90% or less	81%	43%	79%	46%
89% or less	73%	50%	75%	47%
88% or less	60%	59%	71%	47%
87% or less	35%	80%	67%	51%
86% or less	34%	85%	68%	58%
Bounceback admission among discharged ED patients				
99% or less	98%	5%	96%	10%
98% or less	98%	11%	98%	10%
97% or less	90%	23%	95%	11%
96% or less	82%	42%	96%	13%
95% or less	74%	58%	95%	16%
94% or less	54%	73%	94%	18%
93% or less	28%	83%	92%	15%
92% or less	20%	90%	91%	17%
91% or less	8%	96%	91%	16%
90% or less	2%	98%	90%	10%

*ED*, emergency department.
